# Eccentric exercise before a 90 min exposure at 24,000 ft increases decompression strain depending on body region but not total muscle mass recruited

**DOI:** 10.1113/EP091853

**Published:** 2024-06-26

**Authors:** Frode Gottschalk, Mikael Gennser, Mattias Günther, Ola Eiken, Antonis Elia

**Affiliations:** ^1^ Division of Environmental Physiology Swedish Aerospace Physiology Center KTH Royal Institute of Technology Stockholm Sweden; ^2^ Department of Neuroscience, Experimental Traumatology KI Karolinska Institutet Stockholm Sweden; ^3^ Department of Physiology and Pharmacology KI Karolinska Institutet Stockholm Sweden

**Keywords:** aviation, decompression, decompression sickness, eccentric exercise, high‐altitude, venous gas emboli

## Abstract

**Abstract:**

Eccentric upper‐body exercise performed 24 h prior to high‐altitude decompression has previously been shown to aggravate venous gas emboli (VGE) load. Yet, it is unclear whether increasing the muscle mass recruited (i.e., upper vs. whole‐body) during eccentric exercise would exacerbate the decompression strain. Accordingly, this study aimed to investigate whether the total muscle mass recruited during eccentric exercise influences the decompression strain. Eleven male participants were exposed to a simulated altitude of 24,000 ft for 90 min on three separate occasions. Twenty‐four hours before each exposure, participants performed one of the following protocols: (i) eccentric whole‐body exercise (ECCw; squats and arm‐cycling exercise), (ii) eccentric upper‐body exercise (ECCu; arm‐cycling), or (iii) no exercise (control). Delayed onset muscle soreness (DOMS) and isometric strength were evaluated before and after each exercise intervention. VGE load was evaluated at rest and after knee‐ and arm‐flex provocations using the 6‐graded Eftedal–Brubakk scale. Knee extensor (−20 ± 14%, *P* = 0.001) but not elbow flexor (−12 ± 18%, *P* = 0.152) isometric strength was reduced 24 h after ECCw. ECCu reduced elbow flexor isometric strength at 24 h post‐exercise (−18 ± 10%, *P *< 0.001). Elbow flexor DOMS was higher in the ECCu (median 6) compared with ECCw (5, *P* = 0.035). VGE scores were higher following arm‐flex provocations in the ECCu (median (range), 3 (0–4)) compared with ECCw (2 (0–3), *P* = 0.039) and control (0 (0–2), *P* = 0.011), and in ECCw compared with control (*P* = 0.023). VGE were detected earlier in ECCu (13 ± 20 min) compared with control (60 ± 38 min, *P* = 0.021), while no differences were noted between ECCw (18 ± 30 min) and control or ECCu. Eccentric exercise increased the decompression strain compared with control. The VGE load varied depending on the body region but not the total muscle mass recruited.

**Highlights:**

- **What is the central question of this study?**
  Does exercise‐induced muscle damage (EIMD) resulting from eccentric exercise influence the presence of venous gas emboli (VGE) during a 90 min continuous exposure at 24,000 ft?
- **What is the main finding and its importance?**
  EIMD led to an earlier manifestation and greater VGE load compared with control. However, the decompression strain was dependent on the body region but not the total muscle mass recruited.

## 1 INTRODUCTION

Decompression sickness (DCS) is a well‐known risk associated with exposure to significant reduction in ambient pressure (Conkin et al., 2018). A rapid transition to reduced ambient pressure may result in the formation of gas bubbles in tissue and venous circulation. The latter are often termed venous gas emboli (VGE) (Stepanek, 2002). It is generally accepted that the formation of such bubbles, which can occur in many locations and to varying degrees, is the fundamental cause of DCS (Nishi et al., 2003). The severity of DCS symptoms varies, spanning from modest manifestations like itching and joint pain to more severe ones like neurological deficits (Vann et al., 2011). These symptoms are typically preceded by large numbers of VGE (Eiken et al., 2023; Francis & Mitchell, 2003; Sawatzky, 1991), and thus VGE load is used as an indicator of decompression strain (i.e., risk of developing DCS).

It is generally accepted that bubbles originate from precursor micronuclei: small collections of gas, found in vessels and tissues (Arieli & Marmur, 2011; Vann et al., 1980). Thus, *ceteris paribus*, increasing the number of micronuclei prior to decompression would, theoretically, increase VGE load and concurrently aggravate the susceptibility to DCS. It has been suggested that the incidence of VGE and DCS increases when strenuous exercise dominated by eccentric contractions is performed prior to decompression (Damato et al., 1963; Hughes & Eckenhoff, 1986; Nishi et al., 1982; Vann, 1982; Vann & Gerth, 1995). In this context, we recently demonstrated that eccentric upper‐body exercise performed 24 h prior to a hypobaric exposure aggravates the decompression strain compared with control (i.e., no exercise) (Gottschalk et al., 2023). Although the exact underlying mechanism dictating such responses remains largely unknown, it is likely that eccentric exercise increases the presence of micronuclei.

During eccentric contractions, the muscle is forcibly stretched, resulting in exercise‐induced muscle damage (EIMD) (Lindstedt et al., 2001). This is associated with signs of structural damage to sarcomeres and capillaries (Stauber et al., 1990), consequently initiating an inflammatory response and microvascular hyperpermeability (Hotta et al., 2018). In addition, eccentric contractions are known to disrupt cellular membranes leading to an efflux of myocellular proteins into the systemic circulation (e.g., myoglobin and creatine kinase (CK)) (Peake et al., 2017). Accordingly, the higher VGE load observed during states of EIMD may be due to: (i) micronuclei passing into vessels via vascular hyperpermeability (Hotta et al., 2018), (ii) circulating proteins (e.g., myoglobin, creatine kinase and cytokines) altering the surface tension of existing micronuclei and prolonging their lifespan (Philp et al., 1972; Thorsen et al., 1989; Van Liew & Raychaudhuri, 1997), or (iii) strong muscular contractions causing the formation of new micronuclei at locations of reduced hydrostatic pressures (Harvey, 1945; Unsworth et al., 1971; Whitaker et al., 1945). Regardless of the exact underlying mechanism(s) governing the higher VGE load noted during EIMD (Gottschalk et al., 2023), it is important to note that the eccentric exercise was limited to the upper body only. Accordingly, the question that arises is whether increasing the muscle mass recruited during eccentric work (i.e., whole‐body vs. upper body only) would further exacerbate the decompression strain.

This study aimed to investigate the effect of EIMD and its magnitude on high‐altitude‐induced VGE. It was hypothesized that whole‐body eccentric exercise would lead to a greater VGE load as opposed to upper‐body eccentric exercise alone and control.

## 2 METHOD

### 2.1 Ethical approval

This study received ethics approval from the Swedish National Ethics Review Authority in Stockholm (reference number 2021–05293), and all experimental procedures conformed to the *Declaration of Helsinki*, except for registration in a database.

### 2.2 Participants

Based on our previous work with a similar experimental design to the current study (Gottschalk et al., 2023), a minimum sample size of 11 participants was determined a priori, using α = 0.05, β = 0.85, and an effect size of d*z* = 1.01 (G*power software, Heinrich Heine‐Universität, Düsseldorf, Germany). Accordingly, 11 healthy, non‐smoking adult male volunteers were recruited to participate in this study. All participants were military personnel experienced in diving and/or aviation settings. Their mean (SD) age, body mass index and weight were 36 (15) years, 25 (3) kg/m^2^, and 85 (13) kg, respectively.

Prior to the onset of the experimental sessions, participants underwent a physical examination by a physician, with only individuals who satisfied the inclusion criteria with a clean health record (i.e., no history of cardiorespiratory disorders nor any other health conditions such as epilepsy and diabetes) being included in the study. The participants were thoroughly informed, both in written and oral form, about the study's experimental procedures, potential risks and benefits prior to providing their written consent.

### 2.3 Experimental overview

**FIGURE 1:**
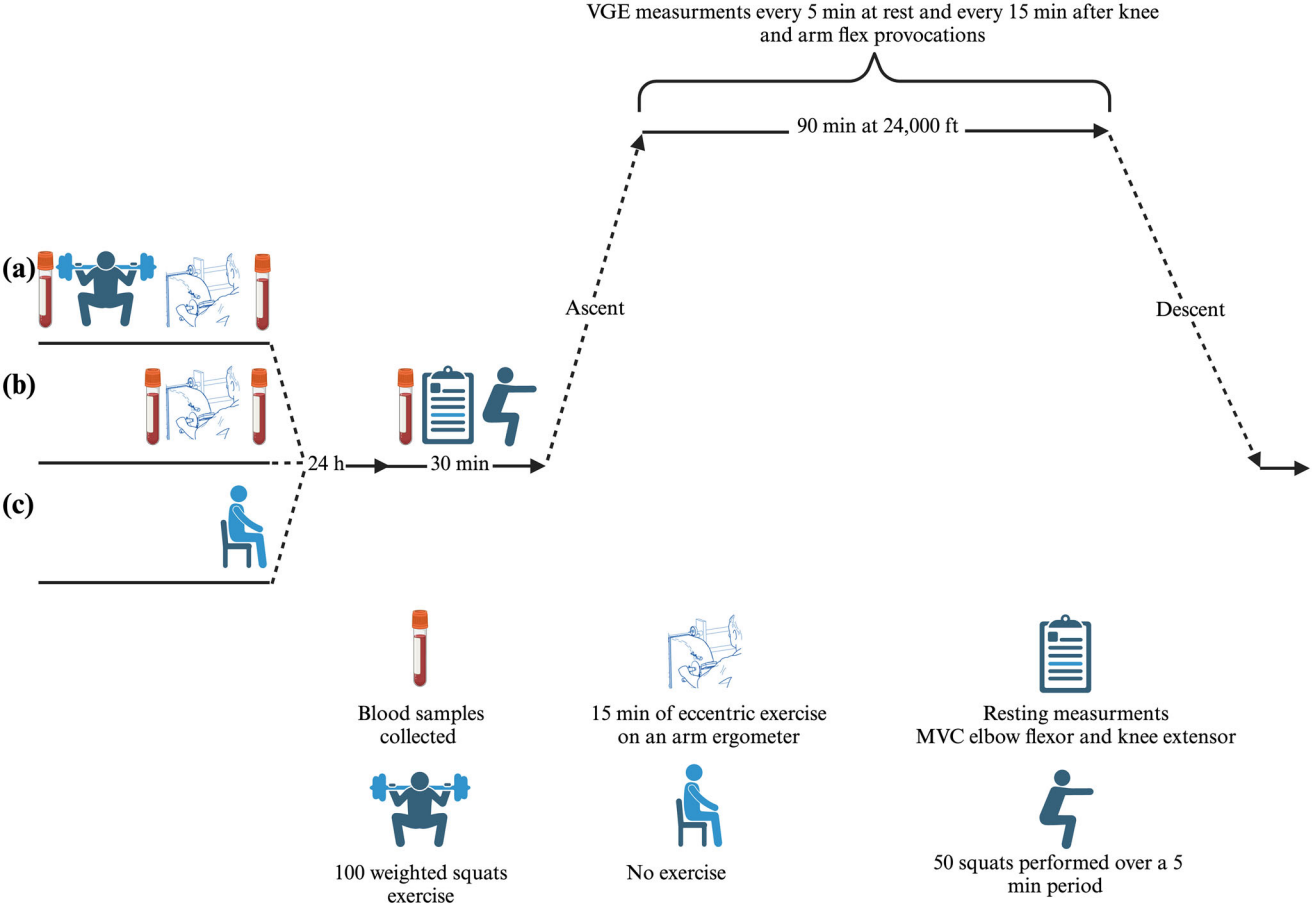
Schematic illustration of the experimental protocol and data collection time points.

All experimental procedures were conducted at the Division of Environmental Physiology of the Royal Institute of Technology, in Solna, Sweden. Participants visited the laboratory on five separate occasions: (i) eccentric whole‐body exercise (ECCw), (ii) eccentric upper‐body exercise (ECCu), and (iii) three times where they were exposed to an altitude corresponding to 24,000 ft for 90 min (Figure 1).

The order of the ECCw and control trials was alternated among participants in a randomized order, with five participants performing first the ECCw. The time interval between these exposures was 22 ± 9 (range: 10–35) days. Due to the repeated bout effect (McHugh, 2003; Meneghel et al., 2014; Nosaka et al., 2001), an interval of 5 ± 3 months (range: 2–9 months) was allowed between the eccentric exercise trials, with the ECCu trial being performed last by all participants.

### 2.4 Experimental protocol

During each experimental visit, the participants reported to the laboratory after a 12 and 24 h abstinence from caffeine‐ and alcohol‐containing beverages, respectively. In addition, the participants were instructed to avoid snus usage for 12 h, to refrain from physical activities for 72 h, and diving or flying for 7 days, prior to and during each experimental session.

Prior to both exercise protocols, the participants underwent a 5 min seated rest period before two 4 mL whole blood samples were drawn from the antecubital vein of the participants’ arm. Thereafter, maximal voluntary contraction (MVC) of the knee extensors and the elbow flexors was measured bilaterally. MVC of the knee extensor and elbow flexor was measured by a force transducer (Binär Teknik AB, Hsintien, Taiwan) at a 90° angle, by maximally contracting the muscles of each corresponding joint isometrically. The experimenter ensured that the participant was standing straight with feet apart and did not lean when initiating the force and then maintained a maximal force for 3 s. Mean peak force derived from the three MVCs were used for subsequent analysis (mean coefficient of variation (CV%) of the knee extensor MVCs and elbow flexor MVCs were 5% and 7%, respectively).

### 2.5 Lower‐body eccentric exercise

The lower‐body eccentric exercise comprised 10 sets of 10 weighted squats, each with a 2 min recovery period between sets (Burt et al., 2013). The bar was loaded with weights equivalent to 60% of the torque generated during the knee extension measurements. With a straight back, legs fully extended and feet hip‐width apart, the bar was positioned on the participants’ shoulders. During the eccentric phase, the bar was lowered until a knee angle of 90° was attained. To emphasize eccentric contractions, the participants were instructed that the downward phase of the squat would last for 2–3 s, whereas the experimenter would assist during the upward phases. If the participants were unable to lift the bar, the experimenter would provide additional assistance so that the targeted 100 squats were reached.

### 2.6 Upper‐body eccentric exercise

The upper‐body eccentric exercise was completed on a custom‐made arm ergometer (Elmer et al., 2013; Gottschalk et al., 2023), with the participants performing 15 repetitions of 1 min work bouts, each interspersed by a 1 min rest. The arm ergometer was driven in a forward direction by a 421 W motor (Transmotec, Taipei, Taiwan), equipped with a torque transducer (Binär Teknik AB, Hsintien, Taiwan) connected to National Instruments modules (National Instruments Corp., Austin, TX, USA) displaying power (W) and total work (N m) that the participants absorbed. The participants were instructed to resist the pedals and maintain a cadence of 50 rpm, and as the first bout began, the output torque was set at 60% of the elbow flexor's MVC (Gottschalk et al., 2023). If the participant was unable to maintain the cadence, the torque output was lowered by 10% each time.

During both exercise interventions, participants were verbally encouraged to perform maximal effort. After completion of the eccentric exercise protocols, MVCs were re‐assessed 5 min after, and two 4 mL whole‐blood samples were drawn 30 min after.

### 2.7 Preliminary measures

The participants reported to the laboratory dressed in sports clothes (i.e., shorts, T‐shirt and sneakers) 24 h after: (i) both the lower and upper body eccentric exercises were performed (whole body eccentric exercise, ECCw), (ii) only the upper‐body eccentric exercise was completed (ECCu), and (iii) avoidance of exercise (Control) (Figure 1). Thereafter, they underwent a 5 min seated rest period after which two 4 mL whole blood samples were drawn. Then, MVC of the knee extensor and elbow flexor were measured, and delayed onset muscle soreness (DOMS) was assessed using the CR10 Borg ordinal scale (Borg, 1998) for each affected muscle group. Then, participants underwent a further 5 min of rest followed by measurements of their heart rate and arterial blood pressure (M3, Omron, Kyoto, Japan). At completion of the resting period, pre‐gelled electrodes were attached on the thorax and neck for impedance and electrocardiography recordings (Physioflow PF07, Enduro, Manatec Biomedical, Paris, France). The participants then performed 50 deep, unloaded knee‐squats over a 5 min period, in an attempt to equalise the participants’ exercise state prior to the altitude exposure.

### 2.8 Hypobaric exposure

Immediately after the 50 deep knee‐squats, the participants entered the hypobaric chamber and were positioned on their left side on a gurney. A pulse oximeter was placed on their index finger to allow for continuous measurement of the capillary oxyhaemoglobin saturation ($S_{pO_{2}}$). Just before (30–60 s) the first pressure drop and for the entirety of the hypobaric exposure, the participants wore a full‐face breathing mask (Poseidon Diving Systems AB, Göteborg, Sweden) and breathed a hyperoxic gas mixture (100% O_2_). During each simulated altitude exposure, the subjects were accompanied by an inside experimenter, who performed the VGE and DCS assessment. The inside experimenter was breathing 100% O_2_ via a full‐face diving mask and a demand valve during, and for 1 h preceding each experiment.

Participants were then exposed to a pressure corresponding to 24,000 ft (7315 m above sea level, ∼40 kPa) continuously for 90 min. This protocol has been found to be successful in provoking the formation of VGE (Ånell et al., 2019; Elia et al., 2021; Gottschalk et al., 2023). Pressure lowering and pressure rise in the chamber was performed at a rate of 5000 ft min^−1^ (1524 m min^−1^). During the 90 min exposure, the participants’ heart rate (HR), cardiac output (CO) and $S_{pO_{2}}$ were monitored continuously. The temperature within the hypobaric chamber was 21 ± 1°C.

### 2.9 VGE and DCS assessment

All ultrasonographic scans and evaluations of VGE were conducted by the same sonographer (Elia et al., 2022) in accordance with the guidelines of Møllerløkken et al. (2016). The prevalence of VGE was evaluated from four‐chamber cardiac images, using an ultrasound system (Philips Ultrasound, CX50, Bothell, WA, USA), equipped with a 1–5 MHz phased‐array‐transducer (S5‐1). The incidence of VGE was assessed at rest (5 min intervals), after three unloaded knee‐flex and three arm‐flex provocations (15 min intervals), performed while the participants remained lying on their left side on the gurney. The knee‐flex provocations were always carried out prior to the arm‐flexes. Enough time (∼30 s) was allowed between the provocations to allow the VGE score to be restored to resting values before the arm‐flexes were carried out. Moreover, symptoms associated with DCS and/or other forms of discomfort were assessed at 15 min intervals, using a 10‐point scale (Borg, 1998). Cardiac images were recorded during a period of 10 cardiac cycles, and the prevalence of VGE was evaluated using the Eftedal–Brubakk (EB) 6‐graded scale (0–5): 0 = no visible bubbles; 1 = occasional bubbles; 2 = at least one bubble every fourth heartbeat; 3 = at least one bubble every heartbeat; 4 = at least one bubble/cm^2^; 5 = whiteout, single bubbles cannot be discriminated. End‐point criteria for any altitude exposure included a single VGE score of 4 or 5, arterial bubbles, and/or symptoms indicative of DCS (e.g., joint pain). VGE assessment was conducted on‐line by the experimenter inside the chamber, and subsequently the recordings were further assessed off‐line. The individual responsible for rating the bubbles in the offline setting was blinded to the conditions, and the offline assessment was used for statistical analysis. The Kisman Integrated Severity Score (KISS) was calculated for each participant according to the following formula:

$$\mathrm{KISS}\;=\frac{100}{4^{\alpha }\left(t_{n}-t_{1}\right)}\;\sum_{i\;=\;1}^{n}\frac{\left(t_{i+1}-t_{i}\right)\left(d_{i+1}^{\alpha }+d_{i}^{\alpha }\right)}{2}$$

where *t_i_* is time of observation in minutes after reaching altitude (for time points 1 to *n*), *d_i_* ultrasound score (grades 0–5) observed at time *t_i_* and α = 3 (the parameter α takes into account that the bubble grade is not a linear measure of bubble quantity) (Kisman et al., 1978).

### 2.10 Blood sample treatment and analysis

After collection, the tubes (Becton, Dickinson & Company, Franklin Lakes, NJ, USA, ref 367862) were gently inverted. For myoglobin, the samples were centrifuged for 10 min in 20°C at 2000 *g* whereas for CK, the samples were spun for 15 min at 1000 *g* at 20°C (Nüve, Kabul, Turkey). Plasma samples were then aliquoted in Eppendorf tubes and were stored at −80°C until subsequent analysis.

CK concentrations were quantified using an activity assay (Sigma‐Aldrich, St Louis, MO, USA, CK, MAK116, CV∼2%) and myoglobin was evaluated using an enzyme‐linked immunosorbent assay (Myoglobin, Abcam, Cambridge, UK, ab171580, CV∼3%).

### 2.11 Statistical analysis

All data were analysed using IBM SPSS Statistics software version 28 (IBM Corp., Armonk, NY, USA). The Shapiro–Wilk test was used to determine whether the data were normally distributed. Mauchly's test of sphericity was used to evaluate sphericity; where the sphericity assumption was violated, the Greenhouse–Geisser correction was implemented. A two‐way (i.e., HR, CO, MVCs of the elbow flexor, CK and myoglobin) and one‐way (i.e., MVCs of the knee extensor) repeated measures analysis of variance (ANOVA) with *post hoc* Bonferroni contrast comparison were used to assess for differences between baseline measurements and other time points. Friedman's test and Wilcoxon's signed‐rank test were used to assess for differences in ordinal data (i.e., peak VGE scores and Borg Scale ratings of DOMS) across conditions. In addition, VGE scores were analysed using a statistical procedure proposed by (Baguley, 2012), whereby the VGE data were rank‐transformed and assigned an ascending rank number. Then, the ranks for the different protocols (i.e., control, ECCw and ECCu) were compared using a one‐way repeated measures ANOVA with *post hoc* Bonferroni contrast comparisons. In addition, VGE scores were converted using the KISS equation (Nishi et al., 2003), and one‐way repeated measures ANOVA with *post hoc* Bonferroni contrast comparisons was used to evaluate differences between protocols. The effect size was evaluated using Cohen's *d* for parametric data and Wilcoxon effect size (*r*) for non‐parametric data. Unless otherwise stated, data are reported as means ± standard deviation (SD) and significance was accepted at *P* < 0.05.

## 3 RESULTS

On eight separate occasions the hypobaric exposures were aborted due to reaching the safety criteria (EB score ≥4). This occurred twice during the control runs (i.e., at minute 45 and 75), three times during the ECCw (i.e., once at minute 60 and twice at minute 75) and three times during the ECCu trials (i.e., all at minute 60). Two participants had to abort all three exposures, and another two participants had to respectively abort ECCw and ECCu. Consequently, inter‐individual comparisons between exposures in these subjects were conducted up to the time point of the earliest aborted exposure.

### 3.1 Exercise and MVC

**FIGURE 2:**
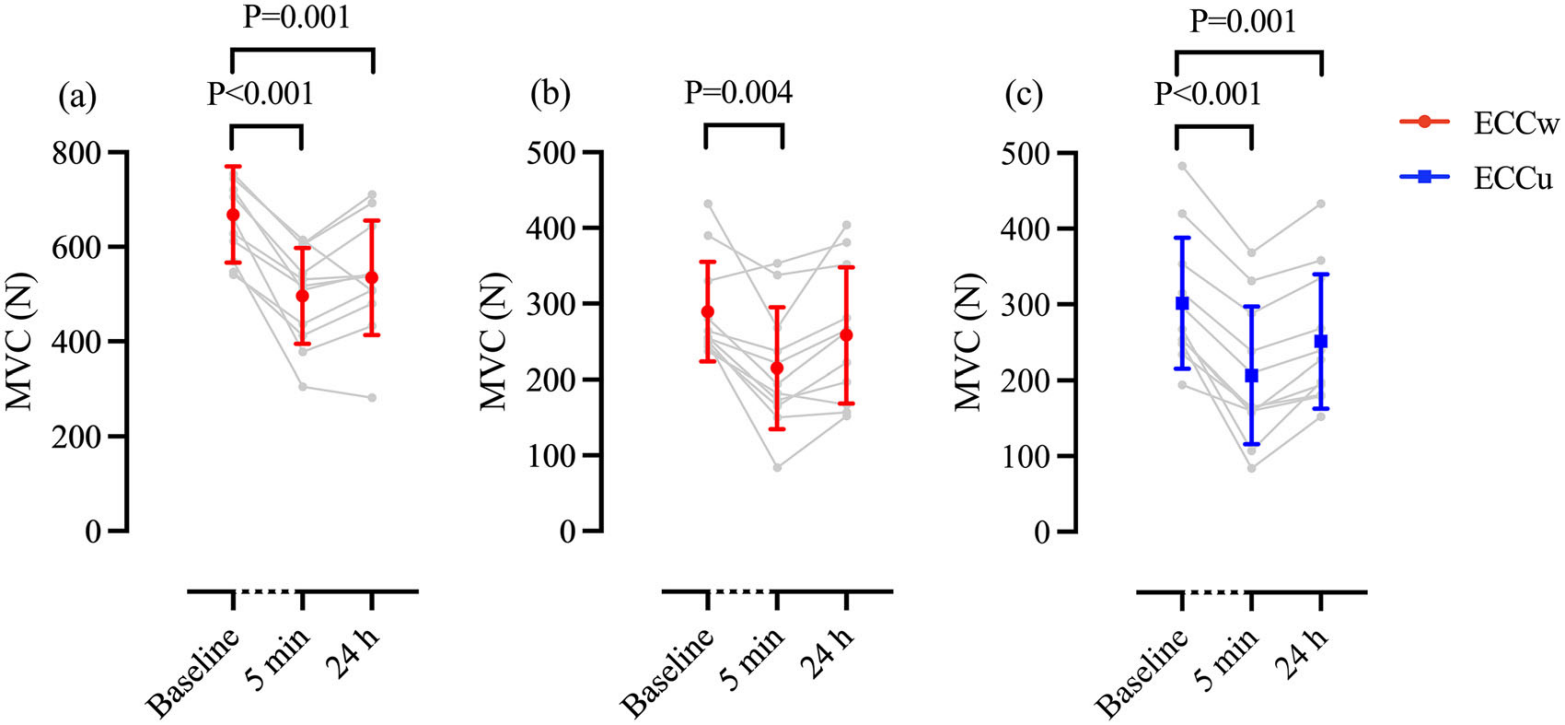
Individual (grey) and mean ± SD (ECCw: red and ECCu: blue) maximal voluntary contraction measurements of the knee extensor (a) and elbow flexor (b, c) at baseline, 5 min and 24 h after the whole‐body (a, b) and upper‐body (c) eccentric exercise protocols. Two‐way (b, c) and one‐way (a) repeated measures ANOVA with *post hoc* Bonferroni contrast; *n* = 11. ECCu, eccentric upper‐body exercise; ECCw, eccentric whole‐body exercise; MVC, maximal voluntary contraction.

A difference in the intensity of eccentric arm cycling was observed between the exercise protocols. Specifically, during the ECCu protocol, participants worked against significantly higher workloads compared to ECCw (248 ± 35 W vs 218 ± 40 W; +15 ± 11%, *P* = 0.004, *d *= 1.309). Baseline MVC measurements (i.e., pre‐exercise or before control altitude exposure) of the elbow flexors did not differ among the three protocols (*P* = 0.596). However, there was a significant difference in MVC measurements between the conditions before chamber exposures (*P* = 0.005). Specifically, the difference was observed between control and ECCw (*P* = 0.038, *d *= 0.916) and control and ECCu (*P* = 0.011, *d *= 1.142), but not between ECCw and ECCu (*P* = 1). After ECCw, the MVC of the elbow flexors decreased from baseline (289 ± 65 N) at 5 min post‐exercise (−27 ± 19%, 214 ± 80 N, *P* = 0.004, *d *= 1.330), but not at 24 h (−12 ± 18%, 258 ± 89 N, *P* = 0.152) (Figure 2). After ECCu, the MVC of the elbow flexors was reduced from baseline (301 ± 86 N) at 5 min (−33 ± 16%, 206 ± 86 N, *P *< 0.001, *d *= 2.455) and 24 h (−18 ± 10%, 251 ± 88 N, *P* = 0.001, *d *= 2.122) (Figure 2). The results of the two‐way ANOVA showed no interaction between the MVC of the elbow flexors following the two exercise interventions for absolute values (N) (*P* = 0.116). By comparing percentage changes, the ECCu protocol revealed a greater reduction in MVC of the elbow flexor 24 h post‐intervention compared to the ECCw protocol (*P* = 0.031, *d *= 0.448).

There were no differences in baseline MVC measurements for the knee extensor between the three protocols (*P* = 0.104). A significant difference was noted in knee extensor MVC measurements between the conditions prior to chamber exposures (*P *< 0.001). Specifically, differences were observed between control and ECCw (*P *< 0.001, *d *= 4.208) and ECCw and ECCu (*P *< 0.001, *d *= 3.351), but not between control and ECCu (*P* = 1). After ECCw, MVC of the knee extensors was reduced from baseline (668 ± 101 N) at 5 min (−26 ± 11%, 496 ± 101 N, *P *< 0.001, *d *= 2.422) and 24 h (−20 ± 14%, 523 ± 129 N, *P* = 0.001, *d *= 1.609) post‐intervention (Figure 2). In contrast, following ECCu, there was no difference in baseline (640 ± 88 N) MVC of the knee extensor at 24 h (−2 ± 5%, 627 ± 90 N, *P* = 0.17). A significant two‐way interaction (*P* ≤ 0.001) was found between the two exercise conditions, with knee extensor MVC significantly lower in ECCw compared with ECCu at 24 h after exercise interventions (*P *< 0.001, *d *= 1.108).

### 3.2 Delayed onset muscle soreness

**FIGURE 3:**
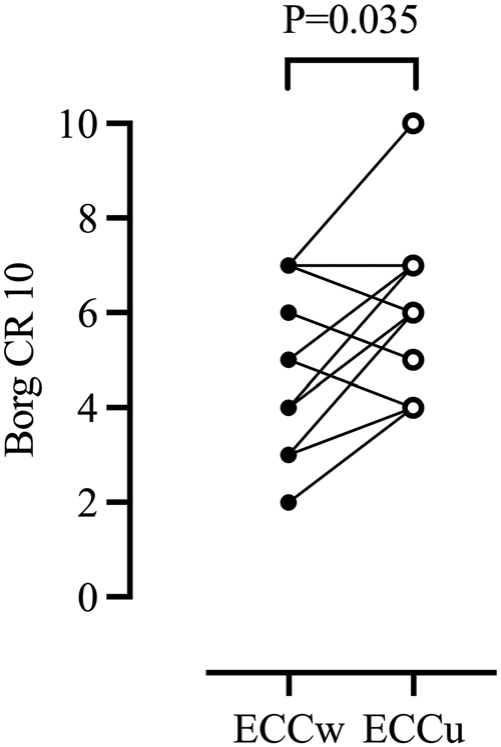
Individual delayed onset muscle soreness scores of the elbow flexors at 24 h after the whole‐body (filled circles) and upper‐body (open circles) eccentric exercise protocols. Wilcoxon signed rank test; *n* = 11. CR, category ratio; ECCw, eccentric whole‐body exercise; ECCu, eccentric upper‐body exercise.

Twenty‐four hours after ECCw, participants reported DOMS in various muscle groups, including the quadriceps, hamstrings and glutes, with a median score of 5 (range 1–8, IQR 2), as well as in the forearms, biceps, triceps, trapezius, shoulders and abdomen, with a median score of 5 (2–7, IQR 3). The highest scores were observed in the knee extensors (median 5, range 3–8, IQR 2) and elbow flexors (median 5, range 3–7, IQR 3). Following ECCu, participants reported DOMS in the forearms, biceps, triceps, trapezius, shoulders, and abdomen, with a median score of 5 (range 3–10, IQR 2). The highest scores were noted in the elbow flexors (median 6, range 4–10, IQR, 2.5). Comparatively, the level of reported DOMS was higher in the elbow flexors after ECCu compared to ECCw (*P* = 0.035, *r *= −0.439) (Figure 3).

### 3.3 Biomarkers

**FIGURE 4:**
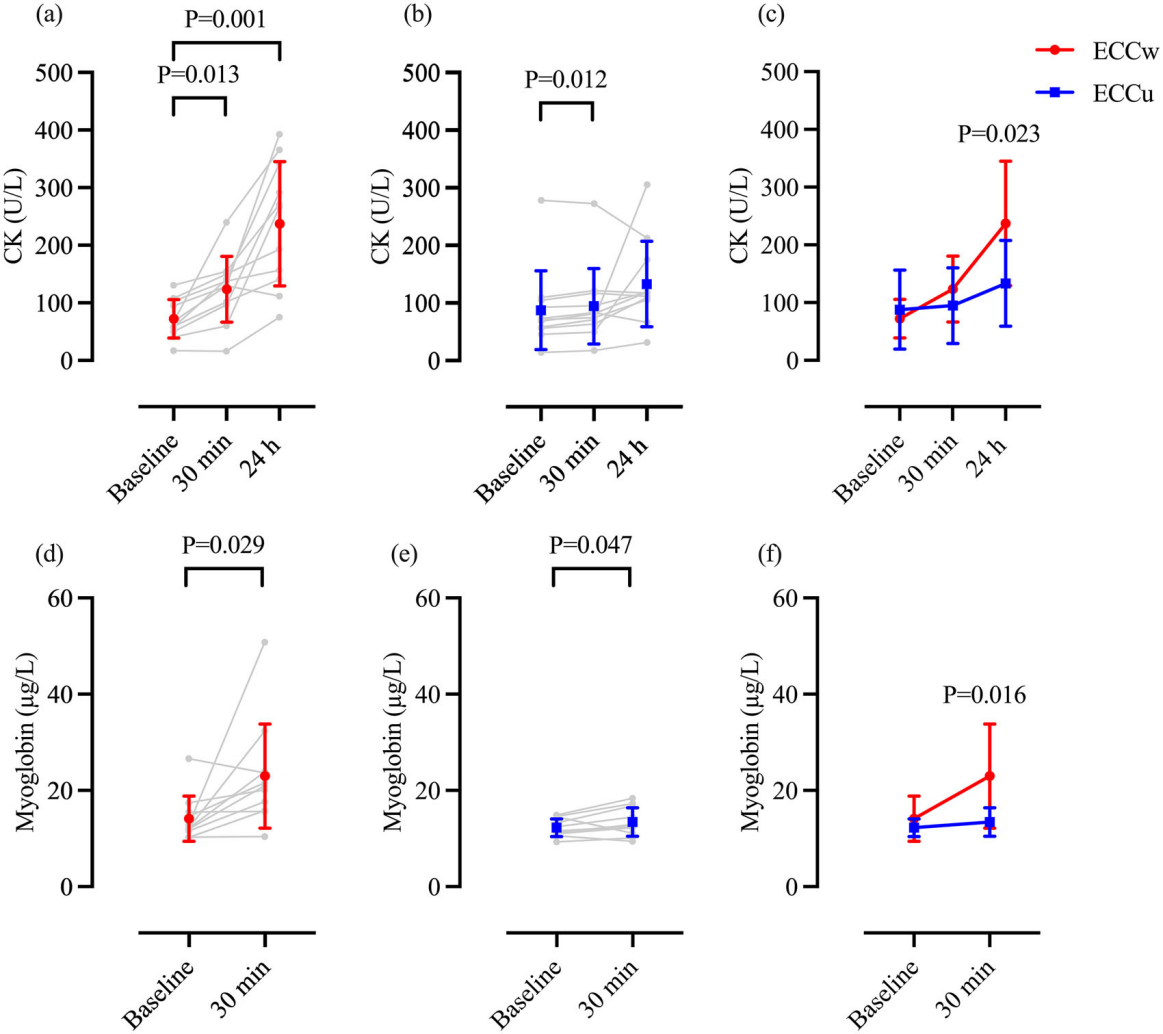
Individual (grey) and mean ± SD (ECCw: red and ECCu: blue) concentrations of CK (a–c) and myoglobin (d–f) from baseline to 30 min (d–f) and 24 h (a–c) after whole‐body (a, c, d, f) and upper‐body (b, c, e, f) eccentric exercise protocols. Two‐way repeated measures ANOVA with *post hoc* Bonferroni contrast; *n* = 11. CK, creatine kinase; ECCu, eccentric upper‐body exercise; ECCw, eccentric whole‐body exercise.

Baseline CK values (pre‐exercise or pre‐control exposure) showed no differences across the conditions (*P* = 0.424). CK levels increased above baseline (72 ± 34 U/L) at 30 min (+76 ± 73%, 123 ± 57 U/L, *P* = 0.013, *d *= 1.108) and 24 h (+227 ± 195%, 237 ± 108 µg/L, *P* = 0.001, *d *= 1.558) (Figure 3) after the ECCw intervention. Likewise, CK levels were higher than baseline (88 ± 69 U/L) at 30 min (+12 ± 7%, 95 ± 66 U/L, *P* = 0.012, *d *= 1.124) but not 24 h (+96 ± 139 %, 133 ± 74 µg/L, *P* = 0.289) after ECCu (Figure 4). In addition, CK concentrations at 24 h were higher after the ECCw than ECCu (*P* = 0.023, *d *= 0.812) (Figure 4).

No differences were observed in baseline myoglobin levels between the three protocols (*P* = 0.188). Following eccentric exercise, myoglobin increased significantly from baseline (ECCw, 14 ± 5 µg/L; ECCu, 12 ± 2 µg/L) at 30 min after the intervention (ECCw, +64 ± 89%, 23 ± 11 µg/L, *P* = 0.029, *d *= 0.794; ECCu, +10 ± 15%, 14 ± 3 µg/L, *P* = 0.047, *d *= 0.548) (Figure 4). Myoglobin concentrations were higher after ECCw compared to ECCu at 30 min after the exercise intervention (*P* = 0.019, *d *= 0.822) (Figure 4).

### 3.4 Venous gas emboli

In three of the participants, no VGE were detected in any altitude exposure. Among the remaining eight participants, VGE were observed in all altitude exposures during the ECCu, in seven during ECCw, and in four during control. A significant difference was noted in VGE scores following arm‐flex provocations across protocols (*P* ≤ 0.001). Specifically, differences were unveiled between control and ECCw (*P* = 0.023, *r *= −0.484), control and ECCu (*P* = 0.011, *r *= −0.543) and ECCw and ECCu (*P* = 0.039, *r *= −0.439) (Table 1). Similarly, using the statistical method proposed by Baguley (2012), a significant difference in maximum VGE score following arm‐flex provocations was discerned between the three protocols (*P* = 0.002). Higher VGE scores were observed in the ECCw (median (range), 2 (0–3), *P* = 0.036, *d *= 0.680) and ECCu protocol (3 (0–4), *P* = 0.011, *d *= 0.817), compared to control (0 (0–2)), while no differences were observed between ECCw and ECCu. There was no difference in maximum VGE scores between the protocols at rest (*P* = 0.190) or following knee‐flex provocations (*P* = 0.154).

**TABLE 1:** Individual and median peak venous gas emboli scores at rest and after knee‐ and arm‐flex provocations for each protocol.

	Control			ECCw			ECCu		
Participant	Rest	Knee‐flex	Arm‐flex	Rest	Knee‐flex	Arm‐flex	Rest	Knee‐flex	Arm‐flex
1	3	3	2	3	4	3	3	3	3
2	0	0	0	1	1	2	2	3	4
3	0	0	1	3	3	3	3	4	3
4	0	0	0	0	0	0	3	3	3
5	3	4	1	1	3	2	1	3	2
6	1	3	0	1	3	2	3	4	3
7	0	0	0	0	0	0	0	0	0
8	0	0	0	1	1	2	1	3	3
9	0	0	0	0	0	0	0	0	0
10	0	0	0	2	2	0	1	0	1
11	0	0	0	0	0	0	0	0	0
Median	0	0	0	1	1	2^*^	1	3	3^*†^
*P*‐value^*^ *P*‐value†						0.023			0.011 0.039

* Significance between exercise protocols and control. †Significance between ECCu and ECCw. Wilcoxon's signed rank test; *n* = 11. Abbreviations: ECCw, eccentric whole‐body exercise; ECCu, eccentric upper‐body exercise.

**FIGURE 5:**
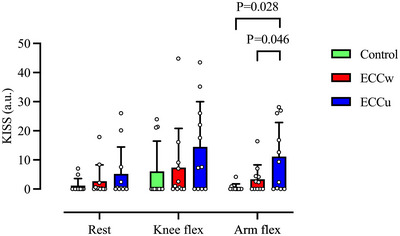
Individual and mean ± SD Kisman integrated severity scores at rest and after knee‐ and arm‐flex provocations for each protocol during the 90 min continuous exposures at 24,000 ft; one‐way repeated measures ANOVA with *post hoc* Bonferroni contrast; *n* = 11. ECCw, eccentric whole‐body exercise; ECCu, eccentric upper‐body exercise; KISS, Kisman integrated severity score.

Significant protocol differences were observed in KISS following arm‐flex provocations (*P* = 0.002). Notably, KISS was higher in the ECCu protocol (11.1 ± 11.7 arbitrary units (a.u.)) compared to control (0.49 ± 1.3 a.u., *P* = 0.028, *d *= 0.966) and ECCw (3.4 ± 4.9 a.u., *P* = 0.046, *d *= 0.760), whereas no difference was found between ECCw and control (*P* = 0.091). There were no differences in KISS during supine rest or after knee‐flex provocations (Figure 5). Additionally, we observed a significant difference in the time taken for the first VGE to be detected between protocols (*P* = 0.015), with an earlier observed VGE during the ECCu protocol (13 ± 20 min) compared with control (60 ± 38 min, *P* = 0.021, *d *= 1.334). There was no difference between ECCw (18 ± 30 min) and control (*P* = 0.098) or ECCu (*P* = 1).

### 3.5 Cardiovascular variables

There were no significant differences between the three conditions in any of the cardiovascular variables (Table 2).

**TABLE 2:** Mean ± SD values for heart rate and cardiac output at rest and after knee‐ and arm‐flex provocations during the 90 min continuous exposures at 24,000 ft.

	HR (min^−1^)			CO (L min^−1^)		
Condition	Rest	Knee‐flex	Arm‐flex	Rest	Knee‐flex	Arm‐flex
Control	63 ± 2	71 ± 2	65 ± 1	5.2 ± 0.9	6.2 ± 1.2	6.3 ± 1.2
ECCw	64 ± 1	74 ± 2	68 ± 2	5.4 ± 1.0	6.5 ± 1.0	6.4 ± 1.4
ECCu	63 ± 2	79 ± 2	66 ± 1	5.0 ± 1.0	6.0 ± 1.5	6.5 ± 1.0
*P*‐value	0.312	0.241	0.251	0.396	0.464	0.499

Two‐way repeated measures ANOVA; *n* = 11. Abbreviations: CO, cardiac output; ECCu, eccentric upper‐body exercise; ECCw, eccentric whole‐body exercise; HR, heart rate.

## 4 DISCUSSION

The aim of this study was to investigate whether the magnitude of muscle mass recruited during the eccentric exercise and the associated EIMD influences the high‐altitude‐induced VGE load. Our results indicate that eccentric upper‐body exercise completed 24 h prior to a 90 min continuous exposure at 24,000 ft, was associated with a higher decompression strain in response to arm‐flex but not knee‐flex provocations, when compared with ECCw and control. Contrary to our hypothesis, eccentric whole‐body exercise did not exacerbate decompression strain compared to upper‐body exercise alone.

Both eccentric exercise protocols resulted in EIMD as noted by the reductions in MVC (Figure 2) and the reported DOMS in the exercised muscles (Figure 3), as well as the increases in muscle injury‐related biomarkers (Figure 4). Notably, MVC reductions in the elbow flexor were evident in both eccentric protocols 5 min after the exercise intervention, with these persisting even 24 h after ECCu but not ECCw. It is also noteworthy that the participants reported higher values of DOMS in the biceps following ECCu compared with ECCw (Figure 3). These differences may relate to the intensity the participants were able to sustain during the arm‐cycling exercise (ECCu: 248 ± 35 W vs ECCw: 218 ± 40 W). Specifically, when the eccentric arm‐cycling was preceded by squats, the participants could not resist as much power, possibly indicating a level of fatigue. An additional explanation is that during the squats, the participants used their arms to stabilize the squat‐bar. As a result, before engaging in eccentric arm‐cycling, these muscles were subjected to a form of warm‐up and stretching, both known to reduce EIMD (Boyd et al., 2023). Taken together, the reductions in MVC and reported DOMS of the elbow flexors suggest that ECCu was associated with a larger effect on the upper‐body muscles compared to that of ECCw.

In agreement with our previous study (Gottschalk et al., 2023), ECCu completed 24 h prior to a hypobaric exposure increased the decompression strain and led to an earlier VGE manifestation compared with control. In addition, a higher VGE load was noted after arm‐flex provocations in both exercise protocols compared to control, with this being greater in ECCu when compared with ECCw (Table 1). Additionally, the ECCu protocol was associated with a higher KISS score following arm‐flex provocations compared to ECCw and control (Figure 5). As mentioned earlier, these differences probably relate to the participants working against a higher resistance during the ECCu (248 ± 35 W) compared with ECCw (218 ± 40 W). Somewhat surprising was the finding that ECCw did not aggravate the VGE load following knee‐flex provocations. Although not directly comparable (i.e., different exercise modality (running) and altitude exposure), our findings are partly in agreement with those of Kumar et al. (1992). In their study, it was shown that performing a 30 min exercise 16 h prior to a hypobaric exposure (21,000 ft) did not affect VGE load when compared with control (i.e., no exercise). Similarly, lower‐body exercise in the form of 150 unweighted squats performed over a 10 min period prior to decompression did not influence the prevalence of VGE when compared with control (i.e., 10 min of seated rest) (Elia et al., 2021). The question that then arises is: what mechanisms underly the present findings?

It is generally accepted that VGE originate from precursor micronuclei (Arieli & Marmur, 2011; Lee et al., 1993; Vann et al., 1980), as *de novo* bubble formation requires very high levels of supersaturation (>100 ATA) (Jones et al., 1999). Therefore, the observation that eccentric exercise increases the release of decompression bubbles suggests that eccentric contractions somehow affect micronuclei. While the mechanistic basis of this effect remains unclear at present, a few options merit considerations. Firstly, eccentric contractions are known to induce structural damage to cells and disturbance to capillaries, consequently increasing microvascular permeability (Hotta et al., 2018). Thus, the increase in VGE could emanate from an increased leakage of micronuclei from the affected muscles to nearby vessels. Yet, whole‐body eccentric exercise, which was associated with a higher EIMD, did not exacerbate the decompression strain. Therefore, this hypothesis seems less likely to be the cause of the higher prevalence of VGE noted in ECCu. An alternative explanation might be that the lifespan of pre‐existing micronuclei may be extended. More specifically, inflammatory cytokines are secreted early during the EIMD recovery period, activating the immune system (Peake et al., 2017). Possibly, a coating of these or other molecules could stabilize the micronuclei, allowing them to persist longer than uncoated bubbles (Philp et al., 1972; Thorsen et al., 1989; Van Liew & Raychaudhuri, 1997). This would theoretically reduce the surface tension of the bubble, allowing for greater influx of nitrogen. However, further research is necessary to prove or refute this line of reasoning.

Another potential mechanism which may be worth considering is the cavitation phenomenon. This is commonly observed in synovial joints when they are stretched beyond their normal range, resulting in a low‐pressure zone, with subsequent bubble formation (Unsworth et al., 1971). By utilizing dual‐frequency ultrasound, signals consistent with microbubbles have been observed following exercise (Wilbur et al., 2010). Thus, micronuclei could potentially be created via negative pressures induced locally in tissue (tendon and muscle attachments) in connection with eccentric muscle contractions. This hypothesis would explain, at least in part, the greater VGE load observed in ECCu. However, if eccentric contractions are capable of inducing cavitation, then why are these responses not also noted after ECCw?

The answer to this disparity may relate to the muscle groups recruited. Although the load during the squats was about double the force exerted during arm‐cycling, the cross‐sectional diameter of the muscles and the tendons differ substantially. For instance, the quadriceps tendon has a cross‐sectional area of 60–100 mm^2^ (Toshiaki et al., 2005) compared with that of the biceps tendon, which is less than 20 mm^2^ (Takeuchi et al., 2021). It can thus be deduced that the biceps tendon experiences a more pronounced negative pressure during eccentric contractions. However, despite the larger cross‐sectional area of the quadriceps tendons, the negative pressures were probably not very different, given that the participants had to lift part of their body weight during the eccentric squat exercise. Whether that holds true for all parts of the muscles cannot be ascertained from our study. Nevertheless, the number of peak eccentric loads was higher in the eccentric arm cycling intervention (50/min × 15 vs. 100). Also, and perhaps, more importantly, despite the presence of DOMS, the lower as opposed to the upper body extremities are more actively recruited during daily life and are subjected to a greater variety of movements. More specifically, the legs are regularly subjected to stepping motions when walking or running, movements that have been shown to reduce the amount of VGE released upon decompression (Blatteau et al., 2005; Dujic et al., 2004). Likewise, studies have demonstrated that whole‐body vibration can lower VGE formation during decompression (Balestra et al., 2016; Elia et al., 2021). The proposed mechanism for VGE reduction in the aforementioned studies is that micronuclei are mechanically dislodged prior to decompression. Consequently, the majority of micronuclei generated during the eccentric squat exercise may have been washed‐out within the 24 h time frame that preceded the hypobaric exposure. Conversely, the DOMS experienced in the upper body led to behavioural changes, with the participants using their arms less (personal communications), potentially preserving any newly generated micronuclei, a supposition that is partly corroborated by the findings of Gennser et al. (2018), whereby extended periods of inactivity and recumbency preceding a hyperbaric decompression were found to increase the formation of VGE.

Considering the repeated bout effect (McHugh, 2003), and its significant variability in duration (i.e., ranging from a couple of weeks to up 6 months) (Foley et al., 1999; McHugh, 2003; Meneghel et al., 2014; Newton et al., 2008; Nosaka et al., 2001), the time interval between our two eccentric exercise interventions was set at a minimum of 8 weeks. The fact that our participants reported higher DOMS and exhibited greater reductions in MVC in their elbow flexors following ECCu (which was always performed as the last intervention) than ECCw suggests that a repeated bout effect was not present. In addition, the higher peak VGE scores recorded after arm‐flex provocations in ECCu, compared to ECCw, offer additional evidence supporting the adequacy of the selected time interval between the two exercise interventions. It is important to point out, however, that our findings are confined to hypobaric decompression, and it is presently unclear whether these responses will be coherently expressed following hyperbaric decompression. Duly, future research should seek to examine the effects of eccentric exercise on VGE load after hyperbaric decompression.

In summary, this study demonstrates that eccentric exercise completed 24 h prior to a 90 min continuous exposure at 24,000 ft increases the decompression strain compared with control; however, the impact of eccentric exercise on VGE formation seems to vary depending on the affected body region, and not the total muscle mass recruited.

### 4.1 Limitations

In the present study, only the order of the ECCw and control was randomized, while all participants completed the ECCu trial last. In future studies one should aim to balance the order of all conditions.

## AUTHOR CONTRIBUTIONS

Frode Gottschalk, Mikael Gennser, Ola Eiken, and Antonis Elia contributed to the conception and design of the research. Frode Gottschalk, Mikael Gennser, Mattias Günther, Ola Eiken, and Antonis Elia conducted experiments. Frode Gottschalk and Antonis Elia performed data analysis. Frode Gottschalk, Mikael Gennser, and Antonis Elia interpreted the results. Frode Gottschalk drafted the manuscript. Frode Gottschalk, Mikael Gennser, Mattias Günther, Ola Eiken, and Antonis Elia edited and revised the manuscript. All authors approved the final version of the manuscript and agree to be accountable for all aspects of the work in ensuring that questions related to the accuracy or integrity of any part of the work are appropriately investigated and resolved. All persons designated as authors qualify for authorship, and all those who qualify for authorship are listed.

## CONFLICT OF INTEREST

None declared.

## Data Availability

Data supporting the study findings may be requested from the corresponding author (F.G) but are not publicly available since they contain information that could compromise the privacy of the research participants.
